# Acute regional changes in myocardial strain may predict ventricular remodelling after myocardial infarction in a large animal model

**DOI:** 10.1038/s41598-021-97834-y

**Published:** 2021-09-15

**Authors:** D. S. Mansell, V. D. Bruno, E. Sammut, A. Chiribiri, T. Johnson, I. Khaliulin, D. Baz Lopez, H. S. Gill, K. H. Fraser, M. Murphy, T. Krieg, M. S. Suleiman, S. George, R. Ascione, A. N. Cookson

**Affiliations:** 1 Department of Mechanical Engineering, University of Bath, Bath, BA2 7AY UK; 2 Department of Translational Science, Bristol Heart Institute and Translational Biomedical Research Centre, Faculty of Health Science, Bristol Royal Infirmary, Level 7, University of Bristol, Bristol, BS2 8HW UK; 3 School of Biomedical Engineering and Imaging Sciences, King’s College London, Westminster Bridge Road, London, SE1 7EH UK; 4 MRC Mitochondrial Biology Unit, The Keith Peters Building, Cambridge Biomedical Campus, Hills Road, Cambridge, CB2 0XY UK; 5 Department of Medicine, University of Cambridge, Addenbrookes Hospital, Hills Rd, Box 157, Cambridge, CB2 0QQ UK

**Keywords:** Cardiovascular biology, Experimental models of disease, Translational research, Prognostic markers, Magnetic resonance imaging, Proteomic analysis, Computational models

## Abstract

To identify predictors of left ventricular remodelling (LVR) post-myocardial infarction (MI) and related molecular signatures, a porcine model of closed-chest balloon MI was used along with serial cardiac magnetic resonance imaging (CMRI) up to 5–6 weeks post-MI. Changes in myocardial strain and strain rates were derived from CMRI data. Tissue proteomics was compared between infarcted and non-infarcted territories. Peak values of left ventricular (LV) apical circumferential strain (ACS) changed over time together with peak global circumferential strain (GCS) while peak GLS epicardial strains or strain rates did not change over time. Early LVR post-MI enhanced abundance of 39 proteins in infarcted LV territories, 21 of which correlated with LV equatorial circumferential strain rate. The strongest associations were observed for D-3-phosphoglycerate dehydrogenase (D-3PGDH), cysteine and glycine-rich protein-2, and secreted frizzled-related protein 1 (sFRP1). This study shows that early changes in regional peak ACS persist at 5–6 weeks post-MI, when early LVR is observed along with increased tissue levels of D-3PGDH and sFRP1. More studies are needed to ascertain if the observed increase in tissue levels of D-3PGDH and sFRP1 might be casually involved in the pathogenesis of adverse LV remodelling.

## Introduction

Myocardial infarction (MI) leads to left ventricular remodelling (LVR), fibrosis, heart failure (HF), and death^1^. Identifying early predictors of LVR may benefit patients.

Changes in LVEF are not reliable in predicting LVR due to confounding factors and do not represent regional changes^2^. It is suggested that changes in LV myocardial strain (MS) may predict LVR^3^, tracking contractility both globally and regionally. Studying global and regional changes in MS post-MI in a relevant experimental model may help with predicting early LVR.

The most effective imaging to measure changes in MS is still debated. Speckle-tracking echocardiography is more common than cardiac magnetic resonance imaging (CMRI)^4^, with 2D speckle-tracking echocardiography featuring comparable spatial resolution to CMRI. However, 3D speckle-tracking echocardiography has a lower spatial and temporal resolution. CMRI is considered the reference method for analysis of LV function and mass. It provides superior image quality with less interference from anatomical structures^5^, and higher reproducibility, especially for circumferential parameters of strain^5,6^.

The efficacy of MS has been investigated in HF with normal or recovered LVEF^7,8^; cardiac amyloidosis and hypertrophic cardiomyopathy. A meta-analysis, pooling 16 studies on HF, acute MI and valvular heart disease, has shown global longitudinal strain (GLS) to be a better predictor of mortality than LVEF, with its prognostic ability surpassing that of radial strain or circumferential strain (CS)^4,9,10^. Yet, MS remains largely a research tool. Longitudinal comparisons of MS between healthy and diseased myocardial territories could predict LVR more effectively, but little has been done in this area, partially due to a lack of relevant pre-clinical models^11^. Consequently, the use of GLS as predictor of late LVR post-MI remains controversial^12^. Global strain measures, calculated as averaged values, may result in loss of sensitivity due to missing key information on regional LV areas, a factor also applicable to measurements of LVEF. However, they can reduce the errors that can be associated with regional measures, with the associated improved reproducibility and ease of explaining their popularity^5^. A regional approach to MS, dividing the myocardium into sections and layers (endocardium vs epicardium), has the potential to be a superior predictor of LVR^3,4,13–15^. However, there are neither established values for healthy or cardiac disease-specific myocardial strains^9,12,16^ nor is there evidence of molecular signatures linked with LVR. Proteomics analysis of the myocardium has ﻿made the evaluation of cellular and cardiac extra-cellular matrix more approachable and reproducible allowing to study molecular signatures in health and disease including LVR^17^.

The aim of this study was to identify changes in MS 1–2 days after acute MI and persisting at 5–6 weeks as signs of early LVR. We also set out to undertake tissue-based proteomics analysis to identify molecular signatures associated with MS and LVR.

## Methods

### Ethical approval for animal procedures

The animal regulated procedures were in line with UK Home Office regulations (Animal Act 1986). The procedures were undertaken under a Project License (No 7008975) granted by the Home Office after formal review and approval by the University of Bristol Animal Welfare and Ethics Review Body (AWERB).

### Myocardial infarction model

Ten Yorkshire female pigs represented the overall cohort of analysis. MI was induced by percutaneous balloon occlusion (60 min) of the mid portion of either the proximal left anterior descending (LAD, n = 8) or the circumflex (Cx) artery (n = 2), according to operator preference. The coronary occlusion was conducted at the mid portion of the targeted coronary arteries, after the first diagonal or the first obtuse marginal branches. Global and regional morphology, function, LV volumes and scar size were assessed by CMRI in all 10 cases at: a. baseline; b. 12–48 h (acute); and **c**. 5–6 weeks (early chronic) post-MI. Previous studies have shown that the immune response post MI can be temporally divided into the early pro-inflammatory phase and the late inflammatory resolution/reparative phase, involving components of both the innate and adaptive immune systems^18^. Left ventricular remodelling was defined as 10% or more changes in left ventricular end-systolic and end-diastolic volumes based on evidence provided by others^19,20^. The additional methods and results for the MI protocol can be found in the Online Supplement.

### Deformation analysis

**Figure 1:**
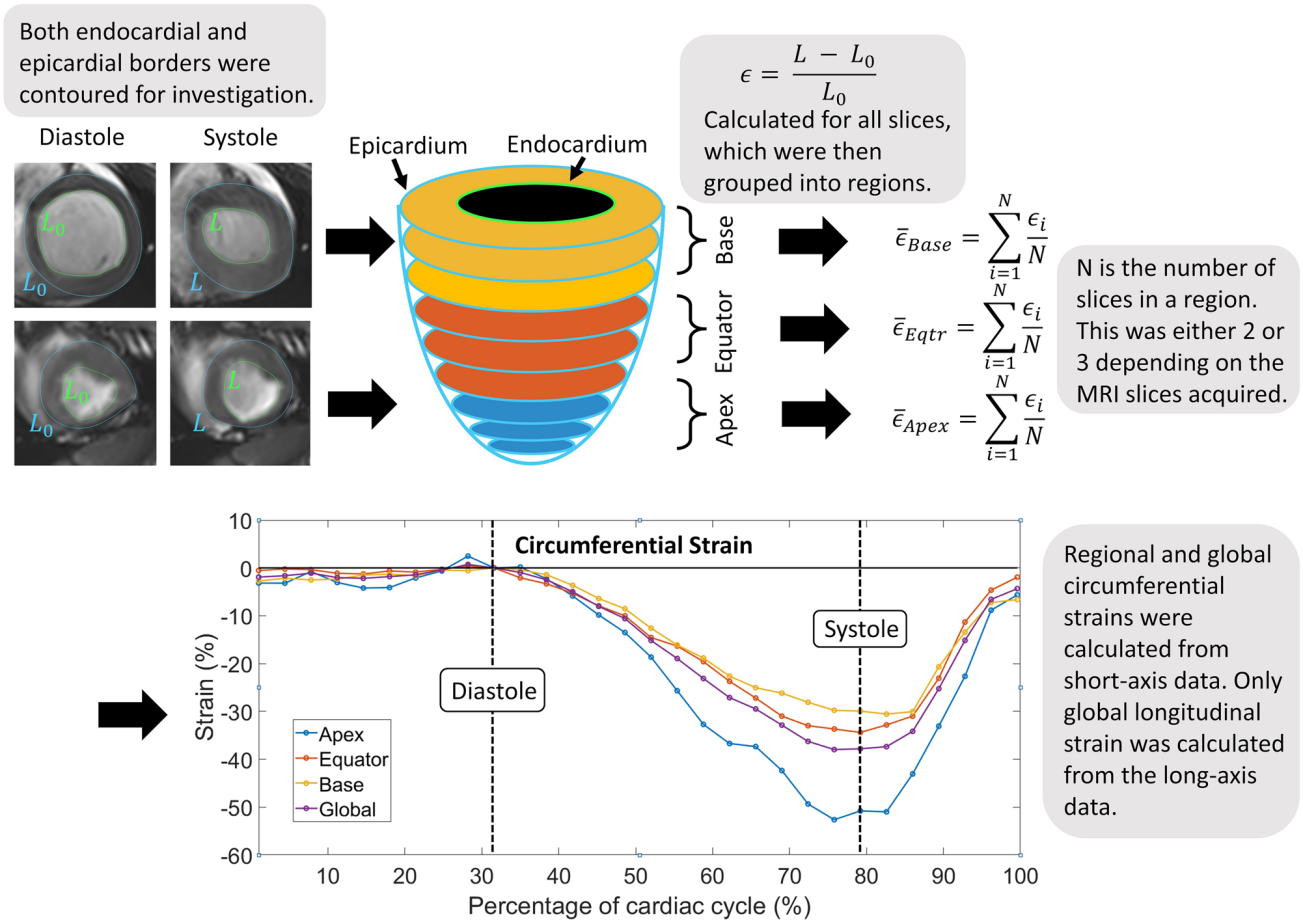
Depiction of workflow from imaging to derived final strains. Short-axis CMRIs with endocardial (green) and epicardial (blue) borders were traced, stacked and grouped into three regions: base, equator/mid, and apex. Strain was calculated for all slices, and the mean was then found by averaging strains in their regions. Finally, the resultant regional and global circumferential strains were found for each time point.

The strain-based metric used in this study was formulated to be robust and reproducible across sites/users/software, use a transparent, non-proprietary algorithm, and be sufficiently sensitive to characterise local ventricular function in a layer-wise manner, as recently reported by our group^21^. The methodology is summarised on the LV schematic in Fig. 1. Slice by slice circumferential strains,$\epsilon ,$ for the endocardium, were calculated throughout the cardiac cycle for each specimen at each time point, according to Eq. (1).

1 $$  \epsilon  = \frac{{L - L_{0} }}{{L_{0} }} $$

The reference length, L_0_, was the endocardial perimeter length at end-diastole, and this was then compared to the endocardial perimeter length, L, throughout the cardiac cycle for a given CMRI slice. Between seven and nine short-axis slices were contoured depending on the specimen and time point; for example, the degree of eccentric hypertrophy and specimen growth could increase the number of slices for a given specimen over time. Strain rate, $\dot{\epsilon }$, as defined by Eq. (2), was also calculated for the circumferential direction, where $L(x,t_{n} )$ and $L(x,t_{n + 1} )$ are endocardial lengths on one cine MRI stack ($x$) at consecutive points in time ($t_{n}$), $\Delta t$ is the time between successive images, and $L_{0} \left( x \right)$ is the end diastolic length for that cine slice.

2 $$   \begin{array}{*{20}c}    {\dot{\epsilon } = \frac{{L\left( {x,t_{{n + 1}} } \right) - L\left( {x,t_{n} } \right)}}{{L_{0} \left( x \right)\Delta t}}}  \\   \end{array}   $$

The same short-axis slices were also divided into three ‘vertical regions’: apex, equator/mid, and base with two or three slices per region, and regional circumferential strains and strain rates were calculated. Similar analyses were conducted for the epicardium. To assess statistically significant changes through time, peak strain and strain rate values were measured. The strain values were averaged over 2–3 slices per region rather than from only one slice as reported by others^2,22^ . Accordingly, the global CS defined here is the average of 7–9 regional slices, rather than of only 3 slices reported by others^22^.

Four-chamber long-axis data were used to investigate LV longitudinal strains and strain rates in both the endocardium and epicardium. Serial volumes were calculated for LV end-systole (LVESV) and end-diastole (LVEDV) by multiplying the area within the endocardial contour for a given slice by the slice thickness, and then all slice volumes were summed to give the ventricle blood pool volume. In addition, serial LV end-systolic volumes indexed to weight (LVESVi) were also calculated by dividing the end-systolic volume by the body surface area (BSA) of the animal. BSA was found through the relation suggested by Kelley et al. shown in Eq. (3)^23^.

3 $$ BSA \left( {m^{2} } \right) = 0.0734*Weight\left( {kg} \right)^{0.656} $$

The endocardial and epicardial contours were manually traced on all short and long axis images in the commercially available software package OsiriX (Pixmeo, Geneva, Switzerland), by one experienced user, and checked by another. Contours were analysed and strain & strain rate values were calculated using an in-house MATLAB script (Release 2017b, The MathWorks, Inc., Natick, Massachusetts, United States).

### Myocardial proteomics and serial troponin I release

Proteomics analysis of infarcted versus remote viable myocardium was performed in five animals in keeping with established methods^24^. Tissue homogenization was obtained with ceramic beads with Ripa Buffer and a protease and phosphatase inhibitors cocktail. The BCA method was used to quantify the protein concentration and samples were prepared at 2 mg/ml for the mass spectroscopic analysis (MSA). Serial troponin release was measured at baseline, reperfusion, 10, 20, 30 min, 1, 4, and 24 h after reperfusion, and before termination. Additional details are available in the Online Supplement.

### Statistical analysis

Non-parametric analysis was performed. Variables are presented as medians and confidence intervals. LVEF, peak values of global LS, global and regional CS, and peak values of corresponding strain rates measured at acute and chronic time-points post-MI were compared with the baseline data using a Kruskall–Wallis test. Observed significant differences were analysed further by using Mann–Whitney U tests. A *p* value of < 0.05 was considered statistically significant, but due to the high number of hypotheses tested, Bonferroni corrections were performed which suggested a *p* < 0.0024 as statistical significance. One-way ANOVA was used for initial assessment with Gabriel’s test to find differences between pairs of means. Linear regression and correlation analyses were performed to assess relationships between the scar weight and other mechanical properties, and between biomarker expression and strain. In the latter case, we elected to focus only on identified proteins showing an R^2^ ≥ 0.95. Statistical analyses were performed in IBM SPSS (IBM Corp. Released 2015).

### Proteomics data analysis

For each protein, an abundance ratio between infarcted and non-infarcted samples was calculated. Proteins found to be at least twofold more expressed in the infarcted myocardium were correlated with the endocardial strain data of the acute phase; a series of univariate linear regression models were performed to correlate each identified protein to each mechanical variable. This statistical analysis was conducted using R version 3.4.4 (R Foundation for Statistical Computing, Vienna, Austria. URL https://www.R-project.org/). For western blotting analysis quantification of band intensity was performed using AlphaEase v5.5 software (now AlphaEaseFC V, https://alphaeasefc-v.updatestar.com/en) followed by background subtraction and correction for protein loading. For evaluation of the differences between the protein expression in the non-ischaemic and ischaemic myocardium, the two-tailed unpaired Student's t-test was used.

### Ethics

The study was carried out in compliance with the ARRIVE guidelines. The procedures were undertaken at the University of Bristol Translational Biomedical Research Centre in accordance with the United Kingdom Animal (Scientific Procedures) Act, 1986 (Home Office Project Licence No 7008975) and the European Union Directive 2010/63/EU. Female Yorkshire pigs (n = 10; weight 62.3 kg ± 5.55 kg) were used in the study. Regulated procedures were in line with Home Office (Animal Act 1986) as described in approved PPL 7008975.

### Patient and public involvement

This research was done without patient involvement. Patients were not invited to comment on the study design and were not consulted to develop patient relevant outcomes or interpret the results. Patients were not invited to contribute to the writing or editing of this document for readability or accuracy.

## Results

Animals were 5–6 months old. Weight range was 55–70 kg, median 62.5 kg at the time of MI and 72–92 kg, median 84 kg at termination.

### Characterisation of MI by CMRI (LVEF, scar size, LVESV and LVEDV) and serial troponin I release

**Figure 2:**
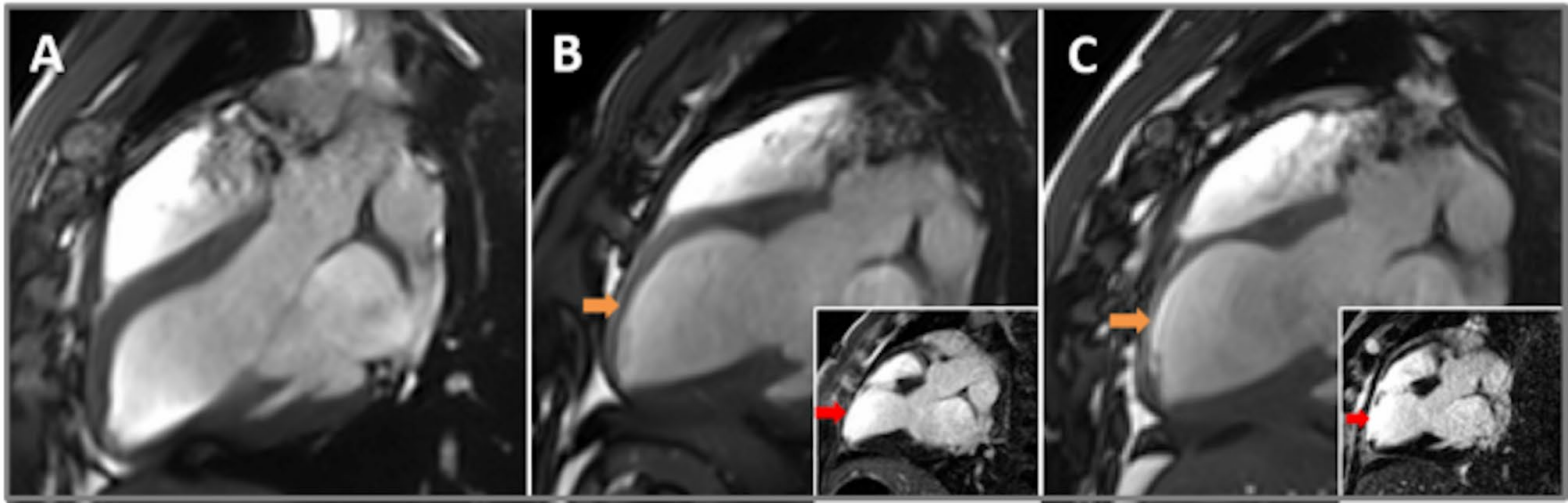
Representative longitudinal CMRI imaging of failing Left Ventricle. From left to right showing CMRI images from the same animal at baseline (**A**), acute (**B**) and chronic (**C**) time-points. Large images show stills of cinematic imaging in 3-chamber view orientation at end diastole demonstrating progressive thinning of the mid to apical antero-septal wall (orange arrows). Inset images show corresponding late gadolinium enhancement imaging demonstrating full thickness late gadolinium enhancement (red arrows).

CMRI outcome is shown in Table S1, Online Supplement. Overall mean LVEF dropped from 56.6% ± 2.5% at baseline to 45.3% ± 7.6% at 4 to 72 h (acute) and to 49% ± 4.6% at 5–6 weeks (chronic). Mean LV scar size was 16.9 g ± 9.1 g at the acute time point and 9.4 g ± 5.6 g at the chronic time-point. Mean LV end-diastolic volume (LVEDV) increased from 131 ± 11.3 mL at baseline to 144.3 ± 5 mL at 12–48 h and to 194.6 ± 27.6 mL at 5–6 weeks post-MI suggesting occurrence of significant LVR over time. Mean LV end-systolic volume (LVESV) increased from 56.8 ± 4.9 mL at baseline to 77.8 ± 16.4 mL at 12–48 h and to 100.4 ± 17.7 mL at 4–6 weeks. Representative longitudinal CMRI scans from the same experiment are reported in Fig. 2 A-C and in Supplemental Videos 1–3). Of the time points assessed, the highest level of troponin I release was recorded at 4 h at 49.6 ± 39.71 ng/ml (Table S2, Online Supplement).

### Myocardial strains

Myocardial strains were calculated from all 10 MI experiments (n = 8 LAD territory and n = 2 CX territory). The occurrence of MI in these two different coronary territories caused tissue damage in the apical and lateral LV wall regions respectively. For the majority of the analysis focusing on LV global metrics, such as LVEF, GCS and GLS, myocardial strains from all 10 experiments were included as these indices should be able to characterise the severity of an infarct & the subsequent LV remodelling regardless of the affected coronary territory. LV remodelling, occurring due to myocardial tissue’s response to the imposed occlusion, encompasses changes in ventricular shape, volume, and function throughout the cardiac cycle. Similarly, MRI data from all baseline, pre-MI scans were retained in the analysis. Nevertheless, for, the statistical analysis of myocardial strain changes in ACS at acute and chronic timepoints only data from the 8 LAD experiments were included, as only these cases were expected to determine an MI affecting the apical region i.e. that covered by the ACS metric. The same approach was taken for the other regional strains ECS and BCS. An evaluation of the regional strains for the CX territory was not performed, because with only n = 2 experiments in this sub-group such an evaluation would not have been meaningful.

### Long-axis global and transmural LV strains

Changes in myocardial strains over time for all animals are shown in Table 1, and Table S3 (Online Supplement). Assessment of endocardial and epicardial GLS did not differ at the acute or chronic time-points versus baseline strain. Endocardial and epicardial strain rates did not differ at the acute or chronic time-point compared to baseline values. No correlations were observed between scar weight and GLS or strain rate.

**Table 1:** Changes in myocardial strains over time.

Parameter	Baseline	95% CI		Acute MI	95% CI		*p* value	Chronic MI	95% CI		*p* value
		Upper	Lower		Upper	Lower			Upper	Lower	
Endocardial ACS (%)	− 37.5	− 31.2	− 49.4	− 17.7	− 16.8	− 27	0.004	− 19.1	− 12.3	− 26.3	0.002*
Endocardial GCS (%)	− 34.9	− 30.5	− 28.4	− 23.8	− 21.1	− 26.1	0.002*	− 27.7	− 17.8	− 31.6	0.006
Endocardial GLS (%)	− 24.7	− 19.9	− 28.9	− 18	− 14.8	− 22.7	0.008	− 21.7	− 12.8	− 27.6	0.171
LVEF (%)	57	52	62	45	40	50	0.002*	50	46	54	0.019
LVESVi (ml/m^2^)	35.9	26.8	49.1	52.1	45.3	60.3	0.045	50	37.4	63.3	0.127

CI = Confidence interval; ACS = Apical circumferential strain; GCS = Global circumferential strain; GLS = Global longitudinal strain; LVESVi = Left ventricular end-systolic volume index; *p* values are for differences compared to baseline results. *denotes changes significant to *p* < 0.0024.

### Short-axis global and regional LV strains

**Figure 3:**
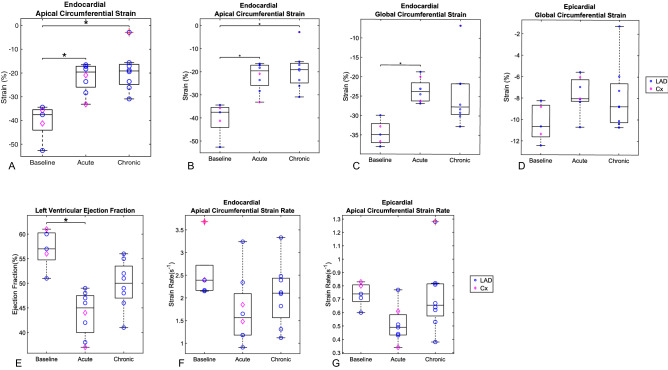
(**A**-**D**) Overtime changes in apical and global circumferential strains. (**A**) Endocardial apical circumferential strain (ACS); (**B**) Epicardial apical circumferential strain (ACS); (**C**) Endocardial global circumferential strain (GCS); (**D**) Epicardial global circumferential strain (GCS). The two data points at the chronic time point with a red cross (in endocardial ACS and GCS) represent outliers greater than the third quartile plus 1.5 times the interquartile range. (**E**–**G**): Overtime changes in LVEF, endocardial and epicardial ACS rates. (**E**) Left Ventricular Ejection Fraction (LVEF); (**F**) Endocardial apical circumferential strain (ACS) rates; (**G**) Epicardial ACS rates*.* The asterisk denotes changes considered significant with *p* < 0.0024. Statistical test: Mann–Whitney (* = identifies significant difference)”.

Endocardial global CS (GCS) and apical CS (ACS) are shown in Fig. 3A-D. Endocardial ACS decreased significantly at the chronic time point (− 19.1%, *p* = 0.002) point compared to baseline (− 37.5%), whereas the change at the acute time point did not reach statistical significance (− 17.7%, *p* = 0.004) (Table 1). GCS showed a significant change at the acute time-point (− 23.8%, *p* = 0.002) vs. baseline (− 34.9%), whereas the change observed at the chronic time-point did not reach statistical significance (− 27.7%, *p* = 0.006). The endocardial equatorial CS (ECS) and basal CS (BCS) strains showed no statistical changes across the two time-points, with all *p* > 0.01 (Table S4, Online Supplement). No significant changes were seen in epicardial CS, with all *p* > 0.03 (Table S4). Endocardial and epicardial strain rates did not differ at the acute or chronic time-points vs. baseline (*p* > 0.008; Fig. 3E–G and Table S4). No significant correlations were observed between scar weight and circumferential strains. The intra-observer variability for LVEF and LVEDV were 3% and 2% respectively, and the inter-observer variation were 8% and 7% respectively.

### LV global function and associations with strain

**Figure 4:**
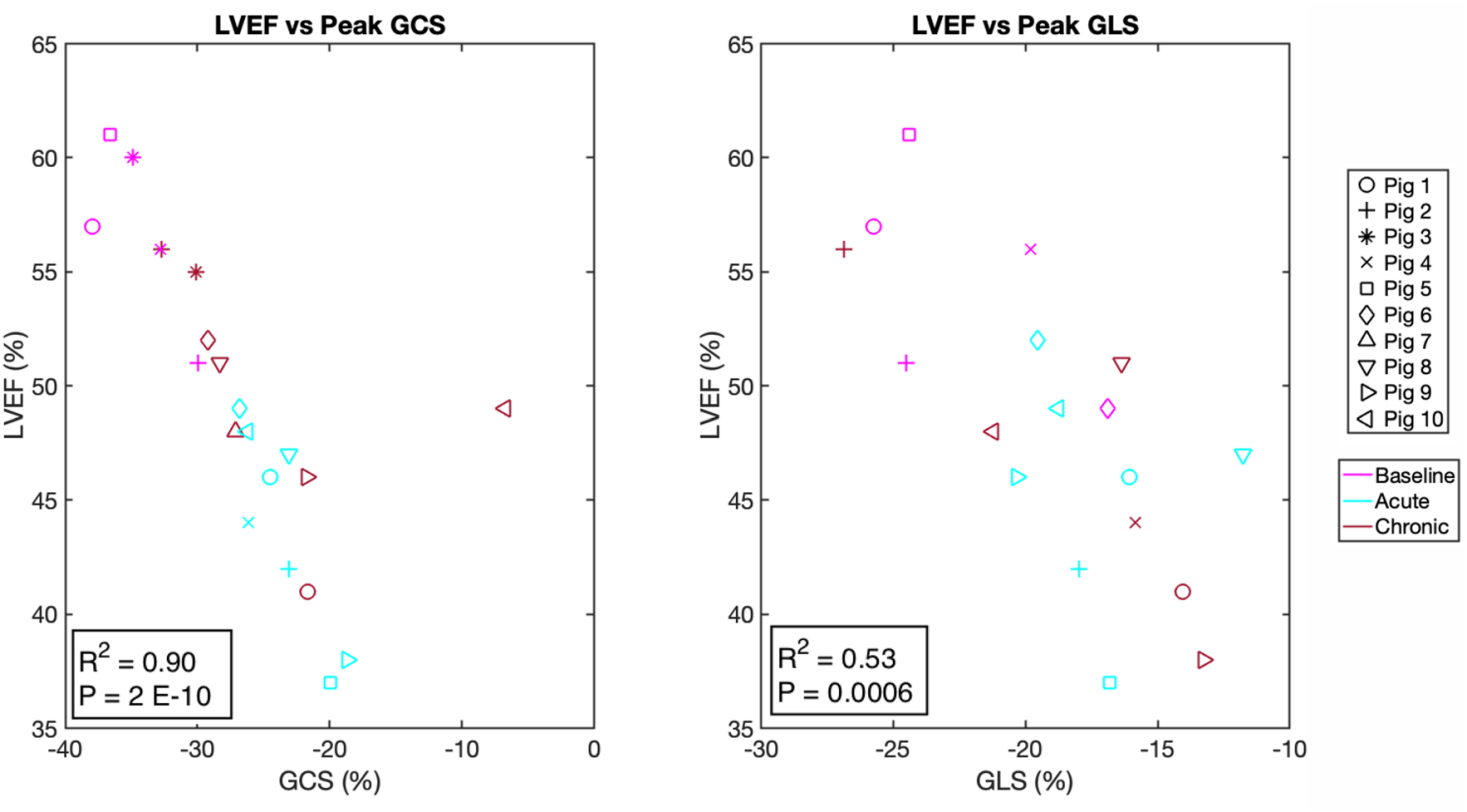
Correlation between endocardial GCS and GLS with LVEF. Scatterplot between endocardial GCS and LVEF (**A**) and between endocardial GLS and LVEF (**B**). Each individual porcine specimen is denoted by a different marker and three different line colours are used to indicate the experimental time point of baseline, acute, and chronic.

LVEF and left ventricular end systolic volume index (LVESVi) were measured by established CMRI methods (Table S1, Online Supplement) with relevant strain contours derived by hand (Table 1). LVEF dropped significantly only at the acute time-point vs. baseline (*p* = 0.0023) (Table 1 and Fig. 3E–G). Scatterplots of GCS versus LVEF (R^2^ = 0.90, *p* < 0.0001) and GLS versus LVEF (R^2^ = − 0.53, *p* = 0.0006) indicated strong and moderate correlations respectively (Fig. 4). LV volumes indexed to weight were assessed given the substantial weight gain observed from acute to chronic time-points. This showed that there were no significant changes in LVESVi from acute to chronic time-points (Table 1). No difference was seen in LVEF values between those measured by CMRI and the relevant strain contours derived by hand. Early changes in strain predicted late LVR: ACS: baseline: − 37.5%, chronic: − 19.1%, *p* = 0.002; GCS: baseline: − 34.9%, acute: − 23.8%, *p* = 0.002, chronic: − 27.7%, *p* = 0.006. Correlation analysis of GCS, GLS, ACS with LVEF and LVESVi are shown in Table S6.

### Myocardial proteomics, LV strains, and validation of D-3PGDH and sFRP1 by western blotting

**Figure 5:**
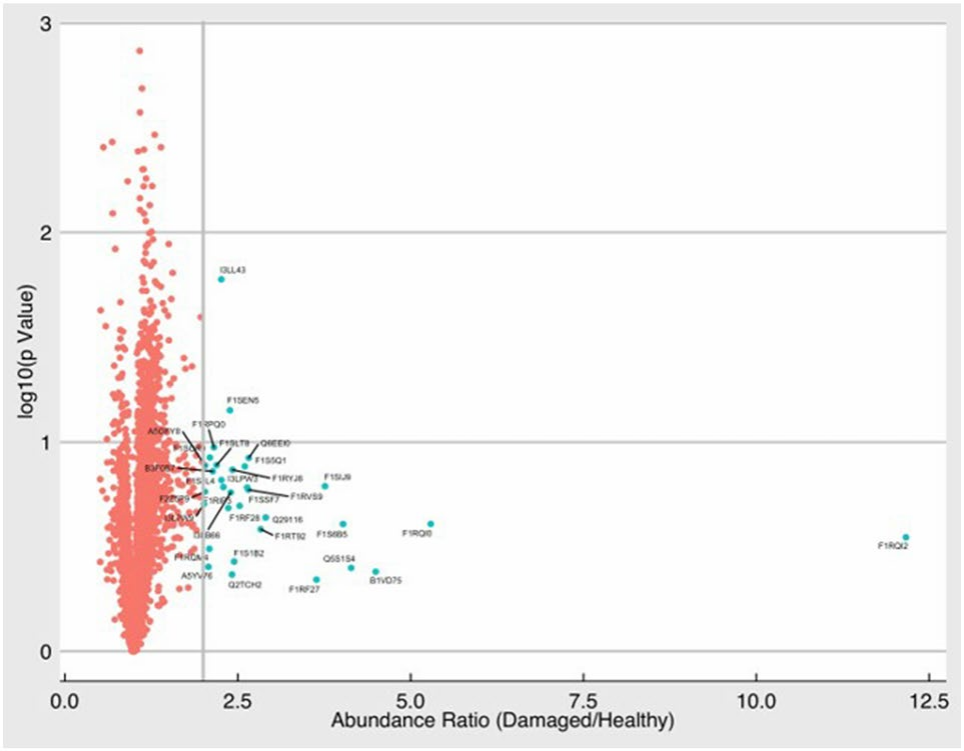
Volcano plot representation of proteomics. Abundance ratios for changes in each protein are shown as log10 of *p* value of infarcted/health segments within the same hearts (n = 5) (R Software version 3.4.4, R Foundation for Statistical Computing, Vienna, Austria. URL https://www.R-project.org/).

**Figure 6:**
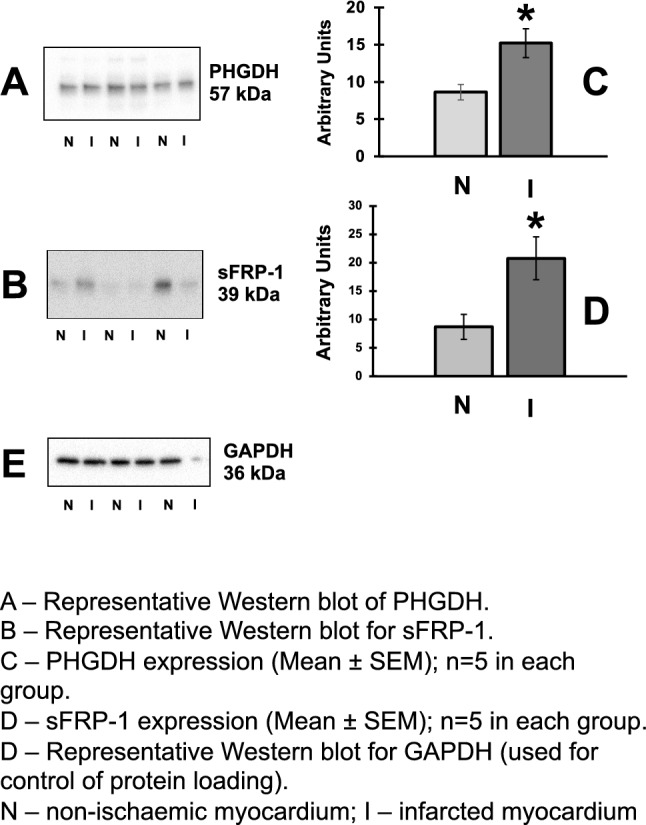
Quantification of D-3PGDH and sFRP1 proteins by western blotting. All data presented as Mean ± SEM; n = 5 in each group. Quantification of D-3-phosphoglycerate dehydrogenase (D-3PGDH) and secreted frizzled-related protein 1 (sFRP1) in lysates of the infarcted myocardium (I) and non-infarcted myocardium (N). (**A**) Representative western blot of D-3PGDH; (**B**) Densitometric quantification of D-3PGDH; (**C**) Representative western blot for sFRP1; (**D**) Densitometric quantification of sFRP1 expression; (**E**) Representative western blot for anti-glyceraldehyde 3-phosphate dehydrogenase (GAPDH, used for control of protein loading); *P < 0.05 versus non-ischaemic myocardium. MW: molecular weight. Statistical Test used: Mann–Whitney. (Figure S1 shows the full blots for the proteins shown in this figure).

Proteomics data and correlation with LV strains are reported in Fig. 5, Tables S5, S6 and S7 (Online Supplement). 5981 proteins were identified: proteins were regarded as differentially expressed if they revealed more than twofold changes in the infarcted area. 39 proteins were at least twice overexpressed in infarcted territories compared to non-infarcted regions (Fig. 5). For the analysis correlating proteomics with strains, proteomics data from 4 hearts was used as strains were not available for the 5 experiments. Significant linear correlations were found between endocardial circumferential strain rate (ECSR) and 21 of the proteins increased in the infarcted territories (Table S6, Online Supplement). The proteins showing the strongest correlation (R^2^ ≥ 0.95) with the ECSR were: D-3-phosphoglycerate dehydrogenase (D-3PGDH, R^2^ = 0.96, *p* = 0.01), cysteine and glycine-rich protein-2 (CG-RP, R^2^ = 0.95, *p* = 0.02), and secreted frizzled-related protein 1 (sFRP1, R^2^ 0.96, *p* = 0.01). Western blotting for D-3PGDH and sFRP1 confirmed that the level of D-3PGDH and sFRP1 protein in the infarcted myocardium was significantly increased compared to the non-infarcted myocardium (both *P* < 0.05, Fig. 6). Western blotting for CG-RP showed no difference.

## Discussion

This study identifies an association between change in regional strain soon after MI, early LVR and abundance of D-3PGDH and sFRP1 myocardial proteins. A change in regional ACS was observed (but not in global measures such as GCS, LVEF or GLS) was observed 1–2 days after MI, which persisted 5–6 weeks later predicting early LVR. Additionally, early LVR post-MI was associated with an hyperexpression of 39 myocardial proteins, of which 21 correlated specifically with ECSR, with D-3PGDH and sFRP1 exhibiting the strongest correlation with ECSR.

Our findings also show that the changes in regional strain (ACS) observed 1–2 days post MI accurately reflect the affected myocardial territories.

GLS has been suggested as a predictor of late LVR in STEMI patients^10^ and in an open-chest coronary ligation porcine MI model^11^. It is argued that GLS may predict LVR as the LV apical region affected by ischemia contains more longitudinal fibres, which contributes more to the local contractile performance and are less affected by ischemia^12^. However, the distribution of the circumferential fibres across the LV might reflect changes to longitudinal and circumferential deformations^12^, therefore suggesting that CS metric might add significantly to gauge predictive information on myocardial deformation^25^.

Our data suggests that regional CS might be more sensitive than GLS in predicting late LVR and quantifying LV function. Averaging strains over few slices within a specific LV region, instead of assessing individual slices or global metrics, might boost the reproducibility and robustness of the method. In addition, this approach ensures that small differences in image location (from patient movement or from scans at different times or different patients) determine less bias when comparing longitudinal data. Also, performing strain over smaller volume/regions is associated with less variation, hence with higher potential of identifying smaller changes. Accordingly, CS has been shown to be an effective indicator of MI, marker of LV function, and infarct transmurality^26,27^. These findings, if confirmed, might affect the type and timing of pharmacological and/or mechanical LV unloading approaches post-MI to prevent heart failure^28^.

Correlations were also found between GCS and LVEF as well as between GLS and LVEF. Both GCS and LVEF were calculated using the same short-axis data, and so a strong correlation was expected based on geometrical considerations. Long-axis data was used for GLS, so the correlation found between these parameters suggests that GLS might be able to detect MI and changes to LV function in keeping with findings by others^28^.

The occurrence of MI and related ischemia/reperfusion injury trigger a storm of molecular signalling, cellular remodelling, inflammatory reaction and fibrosis leading to scar formation and LVR^19,29^. Farah and colleagues^20^ defined LV remodelling as an increase of 10% in ventricular end-systolic or end-diastolic diameter, and found a 58% incidence of LV remodelling after an anterior MI compared with other studies. In the Acute Myocardial Infarction Contrast Imaging (AMICI) trial, the term ’reverse REM’ was employed to denote a > 10% reduction in LVESV found at 6 months in 39% of patients following PPCI^30^, being the only independent predictor of 2-year event-free survival. Based on this definition we found that 75% of our experiments had an LV remodeling > 10% at both LVEDV and LVESV at 5–6 weeks at serial CMR.

Binek et al. found that ischaemia triggers changes in the levels of many myocardial proteins, some of which are linked to contractile function or systolic wall thickness^31^. Proteomics analysis in this study showed 39 hyper expressed proteins, 21 of these being strongly correlated with early changes in regional ECSR. The western blotting analysis showed that D-3PGDH and sFRP1 are significantly expressed within the infarcted myocardium. This finding might indicate their involvement in the early changes in ECSR as well as in determining LVR post-MI. D-3PGDH is the key enzyme for the L-Serine biosynthesis pathway that branches from glycolysis. It participates in a metabolic network interlinking folate and methionine cycles to support cell proliferation and an amplification of function has been associated with a pro-oncogenic role^32^. sFRP1 acts as an inhibitor of the Wnt signalling pathway by binding to Wnt proteins and preventing their association with Frizzled receptors^33,34^. Interestingly, sFRP1 protein has been associated with reduced scar size, improved cardiac function and decreased neutrophil infiltration in a mice model of coronary ligation, indicating a protective role of this protein via reduction of post-MI inflammation^34^. This anti-inflammatory role has been suggested by others in rodents but not in pigs. sFRP1 to suppress the Wnt pathway has potential clinical translation for novel therapies aiming to reduce scar size post-MI and warrants further investigation.

There are limitations to this study. The animals did not have atherosclerotic disease, which might have determined a different proteomic profile and a different pattern of LVR. However, the MI size and other CMRI measures were in keeping with what is observed in humans. In addition, we used a model of ischemia/reperfusion injury with an occlusion time of 60 min. It might be possible that in models with permanent coronary ligation, or with much longer ischemic time, the observed results might differ. Furthermore, the animals gained a substantial amount of weight over the study period with a possible confounding effect on scar size, LV volumes and proteomics. However, it has been suggested that the use of CMRI parameters indexed to the weight of the animal can minimise this effect, although this approach is not in keeping with what is done in humans. However, a marked increase in LVESV was observed as early as 1–2 days post MI suggesting that LVR starts soon after MI and that this early change cannot be biased by weight. Another limitation is the lack of troponin I measurement at 6 and 12 h post MI due to these time-points occurring overnight. A relatively small number of animals (n = 10) was used, with strain analyses and proteomics undertaken on sub-groups: non-parametric statistical tests were used to compensate. Another limitation is related to the lack of information on the dynamic proteomics changes that occur after MI: our study design precluded the possibility of also collecting myocardial specimens 1–2 days post MI in the same animals to characterise the dynamic proteomic processes as previously described by other authors^31^. Finally, while our study indicates that early changes in ACS post MI persist at 5–6 weeks along with early signs of LVR, further studies are needed to confirm the association with late chronic LVR.

In conclusion, this study shows that early changes in regional peak ACS persist at 5–6 weeks post-MI, when early signs of LVR are also observed along with increased tissue levels of D-3PGDH and sFRP1. New studies are warranted to ascertain if early changes in ACS post MI could predict late LVR and if the observed increase in tissue levels of D-3PGDH and sFRP1 might be casually involved in the pathogenesis of adverse LV remodelling.

## Supplementary Information

Supplementary Information 1.

Supplementary Video 1.

Supplementary Video 2.

Supplementary Video 3.
