# Supplementary material for: Acute regional changes in myocardial strain may predict ventricular remodelling after myocardial infarction in a large animal model
Source: Sci Rep. 2021 Sep 15;11:18322. doi: 10.1038/s41598-021-97834-y (PMC8443552; doi:10.1038/s41598-021-97834-y)
Supplement: Supplementary file 1 — Supplementary Information 1. [file 41598_2021_97834_MOESM1_ESM.docx]

**Acute regional changes in myocardial strain may predict ventricular remodelling after myocardial infarction in a large animal model**

D.S. Mansell^1*^, V.D. Bruno^2*^, E. Sammut^2^, A. Chiribiri^3^, T. Johnson^2^, I. Khaliulin^2^, D. Baz Lopez^2^, H.S. Gill^1^, K.H. Fraser^1^, M. Murphy^4^, T. Krieg^5^, M.S. Suleiman^2^, S. George^2^, R. Ascione^2†^, A.N. Cookson^1†^

# **Supplemental material**

**Extended Methods**

Serial blood sampling was collected in five experiments at nine time-points to measure levels of cardiac troponin I (cTnI), shown in Table S2. Myocardial specimens were then collected from each heart for histopathology. Additional myocardial specimen from the same heart from infarcted and non-infarcted territories were collected and subjected to proteomics evaluations to identify overexpressed proteins (n=4). These assessed using common assays from standard clinical practice was undertaken at each time point.

*Sample size*

The sample size used reflects similar previous work in pig on myocardial strain by Sun and colleagues( *J Am Soc Echocardiogr. 2007;20(5):498–504*). In their analysis, conducted on 7 pigs, they found that the circumferential strain changed from 13 ± 3 % at baseline to 7 ± 2 % post MI and to 7 ± 2 % at 4 weeks (echocardiographic assessment only and permanent LAD ligation) with a mean difference of 8 % between baseline and postoperative measure (with a SD of 2): based on these figures, in a paired t-test comparison (baseline vs postop), 10 animals are sufficient to have a statistical power of > 90% with alpha error set at 0.05.

*Animal Preparation*

The procedures were undertaken at the University of Bristol Translational Biomedical Research Centre in accordance with the United Kingdom Animal (Scientific Procedures) Act, 1986 (Home Office Project Licence No 7008975) and the European Union Directive 2010/63/EU. The animals received Aspirin 75 mg once daily throughout the experiment. Female Yorkshire pigs (n=10; weight 62.3 kg ± 5.55 kg) were used in the study. Regulated procedures were in line with Home Office (Animal Act 1986) as described in approved PPL 7008975. Briefly, following a week of acclimatisation, animals were anaesthetised according to clinical standard by a veterinary anaesthetist. Animals underwent a baseline CMRI scan, then during the same anaesthesia were transferred to the cath-lab laboratory. Myocardial infarction was created by expert interventional cardiologists using percutaneous balloon occlusion (60 mins) of either the proximal left anterior descending artery (n=8) or the circumflex artery (n=2), guided by X-ray fluoroscopy. The median diameter of the coronary balloons was 3 mm (range 2.5 to 3.5 mm) while the median length was 15 mm (range 12 to 15 mm). The mean pressure of occlusion was 10.6 atm (median 12, range 6 to 14 atm). Animals were then recovered. At four hours to two days (acute, n=10) and 5-6 weeks (chronic, n=10) post MI, under general anaesthesia, animals underwent repeat CMRI, followed by termination, under the same anaesthesia. All animals received amiodarone (300 mg over 4 hours) and magnesium (8 mg over 4 hours) through a central venous line as standard during the MI induction in order to minimise the occurrence of ventricular arrhythmia. Despite these preventive measures, two episodes of ventricular fibrillation and one episode of ventricular tachycardia occurred and were successfully resuscitated by DC cardioversion using external defibrillation. All animals recovered from the MI induction procedures and completed the study according to the predefined protocol. Standard CMRI outcomes were recorded at each experimental stage, summarised in Table S1.

*Anaesthesia*

Premedication was achieved via intramuscular injection of ketamine (10 mg/kg) and Dexmedetomidine (15 mcg/kg); after sedation, oxygenation was granted with an oxygen flow of 5 l/min. The induction of general anaesthesia was achieved via IV infusion of Propofol at 1.0 mg/kg boluses. Anaesthesia was maintained by Isoflurane in oxygen with the vaporiser set at 2%. The animals were mechanically ventilated, and setting were adjusted to maintain ET-CO2 of 35-45 mmHg.

*Myocardial infarction model*

A longitudinal incision was made on the left side of the neck and a central venous line was inserted in the jugular vein to deliver anti-arrhythmic drugs during the procedure. The common carotid artery was then exposed and cannulated: a Judkins Left 3.5 (JL 3.5 – Medtronic Limited, Watford, UK) catheter was used to visualize both coronary arteries and to insert the guidewire and the angioplasty balloon for the occlusion of the target coronary. Serum cardiac troponin I was measured at baseline, reperfusion, 10, 20, and 30 minutes after reperfusion, and at 1 hour, 4 hours, 24 hours, and 5-6 weeks post-MI. After the last CMRI scan, animals underwent median sternotomy to expose the heart with euthanasia involving intracoronary injection of cold cardioplegic solution. Myocardial specimens of LV infarcted and non-infarcted coronary territories from the same hearts (n=5) were subjected to comparative proteomics evaluation followed by western blot validation.

*CMRI protocol and strain calculation*

Multi-slice cine CMRI was acquired in short-axis and in two-, three-, and four-chamber long-axis views. For strain calculation endocardial and epicardial contours of CMRI images were manually traced on all short- and long-axis images in the commercially available software package OsiriX (Pixmeo, Geneva, Switzerland), by one experienced blinded user. Papillary muscles and trabeculae were included in the LV blood pool when contouring following guidelines from the American Heart Association (2). Inter- and intra-observer variability of LVEF and end-diastolic volume were assessed using repeated measurements of endocardial contours from two experienced users. Myocardial scar weight in grams was measured by late gadolinium enhancement using the full width half maximum method.

*CMRI protocol*

Animals were anaesthetised and intubated, then maintained under general anaesthesia by a veterinary anaesthetist with experience in large animal work. Scans were performed with animals in supine recumbence with the surface coil positioned centrally on the sternum. Images were acquired on a Magnetom Prisma^®^ 3T system, (Siemens Healthcare limited, Erlangen, Germany) using the integrated spine coil posteriorly and the Siemens “Body 13” coil anteriorly. Cardiac gating was achieved using a surface ECG or in cases where there was poor triggering, a peripheral pulse monitor placed on the animal’s tail was used. Due to the importance of a stable ECG, the animals were shaved, and the skin prepared with Nuprep^®^, (Weaver and Company, Aurora, CO) to improve skin contact. Breath holds were achieved by temporary interruption of ventilation, controlled by the veterinary anaesthetist with adequate recovery between breath holds. Animals were scanned acutely at 24 hours prior to termination. After acquisition of localiser images, True–FISP functional cinematic images were acquired in the three long axis planes (2-,3- and 4- chamber orientation) of the left ventricle and in a contiguous stack of short axis slices from base (at the level of the mitral valve) to the apex. The acquisition parameters are as follows: slice thickness 8mm, base resolution 208, phase resolution 80%, TE 1.39 ms, TR 46.5 ms, flip angle 45^o^, bandwidth 1335 Hz/Pixel, calculated phases (temporal resolution) 30, data segments per R-R 15. This allowed for the visual assessment of the 17 American Heart Association (AHA) myocardial segments. To minimise off resonance and centre frequency artefacts a True-FISP Frequency Scout was acquired. After reviewing the resulting images, the optimal frequency was subsequently used in the cinematic images. Arrhythmia rejection (acceptance window 200-300ms) was applied where necessary. After cine imaging, a dose of 0.2mmol/kg body weight of Gadobutrol (Gadovist^TM^, Bayer) contrast agent was administered. Early enhancement images were acquired 2 minutes post contrast injection in the three long axis using 2D FLASH, (slice thickness 8mm, base resolution 256, phase resolution 83%, flip angle 15^o^, TE 2ms, TR 750ms, TI 500ms) and the short axis using a single shot 3D FLASH (slice thickness 8mm, base resolution 256, phase resolution 57% slice resolution 70% slice partial fourier 6/8, flip angle 15^o^, TE 1.31ms, TR 700ms, TI 500ms).

Late gadolinium enhancement images were acquired after 8-10 minutes in line with SCMR guidelines. Images were acquired in the long axis and short axis using a 2D FLASH PSIR pulse sequence (slice thickness 8mm, base resolution 256, phase resolution 64%, TE 1.55ms, TR 750ms, TI 300-350ms - based on TI scout images)^11^.

*Image analysis*

Images were analysed using CVI42, v5.6.6, (Circle Cardiovascular Imaging, Calgary, Ontario, Canada) by consensus of two expert readers with several years of experience in CMR and Level III SCMR accreditation. Left ventricular volumes and mass were analysed by manually delineating the ventricular end diastolic and systolic contours in the short axis stack. Papillary muscles and trabeculations were included in blood pool as per SCMR guidelines (Kramer et al JCMR 2013). Left ventricular volumes and ejection fractions were calculated automatically according to Simpson’s rule. Values were normalised to body surface area. Left ventricular mass was measured in end-diastole automatically using the endocardial and epicardial contours. Late gadolinium enhancement was quantified semi-automatically using the full-width half maximum (FWHM) technique. The enhanced volume was calculated and represented as a percentage of the total left ventricular mass. The no reflow volume was traced manually and also represented as an absolute volume and percentage of total left ventricular mass.

*Proteomics analysis and Quantification of D-3PGDH and sFRP1 proteins by western blotting*

Frozen tissue of the porcine ventricles collected from the infarcted and viable areas was placed in a tight-fitting glass homogenizer with RIPA lysis and extraction buffer contained: 150 mM NaCl, 50 mM Tris-HCl (pH 8.0), 1% NP-40, 0.5% sodium deoxycholate, 0.1% SDS, complete protease inhibitor cocktail (Roche Diagnostics) and phosphatase inhibitor cocktail 3 (Sigma). The samples were homogenized, transferred into siliconized tubes, incubated for 10 min to maximize protein solubilization and centrifuged at 17,000*g* for 10 min. All these procedures were carried out on ice or at 4°C. Protein concentration was adjusted to 5 mg·ml-1 using BCA Protein Assay (Thermo Fisher Scientific, Loughborough, UK). Since *Sus scrofa* gene and protein annotation is not complete, the results of the MSA/MSA scans were compared with both *S. scrofa* and *Homo sapiens* protein databases, even though this study focused on *S. scrofa* proteins. The fractionated peptides were analysed by nano-liquid chromatography-tandem mass spectrometry analysis (nanoLC-MSA/MSA) using a Q-Exactive hybrid quadrupole orbitrap mass spectrometer.

The samples were boiled at 100°C for 10 min and then diluted (1:1 v·v-1) with the SDS sample buffer containing: 50 mM Tris-HCl, 2 mM EDTA, 12% glycerol and 10% SDS, 5% 2-mercaptoethanol and 0.01% bromophenol blue. Samples (35 μg per well) were separated by 10% SDS-PAGE and subjected to western blotting with anti- D-3PGDH and anti-sFRP1. Each blot contained samples of both infarcted and viable myocardium to allow direct comparison. Protein loading was evaluated by western blots using anti-GAPDH antibodies.

Western blots for D-3PGDH (Sigma, cat. # HPA021241, diluted 1:1,000) and sFRP1 (Abcam, cat. # ab126613, diluted 1:1,000) was performed using anti-rabbit IgG horseradish peroxidase (HRP) secondary antibody (GE Healthcare, cat # NA934V, diluted 1:10,000). Additional western blots for sFRP1 pathway related Frizzled-1 (Santa Cruz, cat. # sc-398082, diluted 1:500) was performed using mouse IgGκ light chain binding protein conjugated to HRP (Santa Cruz, cat. # sc-516102, diluted 1:5,000). All blots were detected with Amersham™ ECL™ western blot analysis system (GE Healthcare, cat. # RPN2109) or Amersham™ ECL™ Prime western blot detection reagent (GE Healthcare, cat. # RPN2232) and processed using a ChemiDoc™ MP imaging system (Bio-Rad). Quantification of band intensity was performed using AlphaEase v5.5 software followed by background subtraction and correction for protein loading.

**RESULTS: Further Statistics**

Gabriel’s test confirmed that endocardial ACS and LVEF changed significantly from baseline at the acute point (p=0.001 and p=0.00023 respectively), however, it did not show any significant difference between GCS at the acute point when compared to baseline measurements (p=0.009). Examining changes at the chronic time point, Gabriel’s test found that ACS also decreased significantly from baseline results at the chronic time point (p=0.00026), but still with no statistically significant difference in GCS (p=0.02).

**Supplementary Tables**

Table S1: Longitudinal CMRI outcome over time

| Parameter | Baseline  (n=5; mean and SD) | Acute  (n=10; mean and SD) | Chronic  (n=10; mean and SD) |
| --- | --- | --- | --- |
| Swine mass (kg) | 62.3±5.55 | NA | 82.89±6.56 |
| LV Mass (g) | 88.6±6.31 | 89.7±10.8 | 107.62±24.42 |
| LVEDV (ml) | 131±11.3 | 144.3±25.05 | 194.62±27.57 |
| LVESV (ml) | 56.8±4.9 | 77.8±16.4 | 100.38±17.75 |
| LVEF (%) | 56.6±2.51 | 45.1±7.55 | 49.12±4.64 |
| Scar Mass (g) | NA | 16.9±9.1 | 9.38±4.53 |
| Scar Mass (%) | NA | 19.7±11.7 | 9.0.5±5.23 |

SD=Standard deviation; LV=Left ventricle; LVEDV=Left ventricular end-diastolic volume;

LVESV=Left ventricular end-systolic volume; LVEF=Left ventricular ejection fraction.

Table S2: Serum cardiac troponin I (CTnI) concentration at different time-points

|  | Baseline | Reperfusion | After reperfusion | | | | | | Termination |
| --- | --- | --- | --- | --- | --- | --- | --- | --- | --- |
|  |  |  | 10 min | 20 min | 30 min | 1 h | 4 h | 24 h |  |
| CTnI (ng/ml)  (n= 10) | 00.2 ±0.01 | 0.03 ±0.02 | 1.15± 2.01 | 6.53 ±9.61 | 10.82 ±16.93 | 25.52 ±35.79 | 49.66 ±39.71 | 9.61 ± 5.56 | 0.02 ±0.03 |

Table S3: Appendix of mean and medians for all strain, strain rate, and LVEF data

|  | **Baseline** | | | | **4 Hours** | | | | **Chronic** | | | |
| --- | --- | --- | --- | --- | --- | --- | --- | --- | --- | --- | --- | --- |
| **Variable** | **Mean** | **Median** | **CI Lower** | **CI Upper** | **Mean** | **Median** | **CI Lower** | **CI Upper** | **Mean** | **Median** | **CI Lower** | **CI Upper** |
| **GCS endo** | -34.4395 | -34.8878 | -38.3892 | -30.4897 | -23.5610 | -23.7713 | -26.0624 | -21.0596 | -24.7010 | -27.6988 | -31.5660 | -17.8360 |
| **ACS endo** | -40.3249 | -37.5458 | -49.4415 | -31.2082 | -20.1790 | -17.7434 | -23.9859 | -16.3722 | -19.3052 | -19.1286 | -26.2927 | -12.3177 |
| **ECS endo** | -32.5087 | -33.5623 | -36.2143 | -28.8031 | -22.5686 | -21.9692 | -26.3135 | -18.8237 | -24.6822 | -25.9493 | -32.0968 | -17.2676 |
| **BCS endo** | -32.6362 | -33.6379 | -36.6110 | -28.6614 | -29.6299 | -30.5597 | -32.6960 | -26.5637 | -29.6598 | -31.8177 | -38.4780 | -20.8417 |
| **GCSR endo** | 1.9985 | 1.8383 | 1.4016 | 2.5953 | 1.1162 | 1.1594 | 0.9693 | 1.2632 | 1.5559 | 1.2126 | .9657 | 2.1461 |
| **ACSR endo** | 2.5582 | 2.3868 | 1.7631 | 3.3533 | 1.7499 | 1.4145 | 1.0397 | 2.4601 | 2.0803 | 2.1011 | 1.4960 | 2.6647 |
| **ECSR endo** | 2.4624 | 2.4451 | 1.9409 | 2.9839 | 1.4396 | 1.4481 | 1.1238 | 1.7554 | 2.2081 | 1.7632 | 1.2306 | 3.1855 |
| **BCSR endo** | 2.4908 | 2.1805 | 1.4509 | 3.5308 | 1.6421 | 1.6439 | 1.2803 | 2.0040 | 2.1311 | 2.0639 | 1.3320 | 2.9303 |
| **GCS epi** | -10.2934 | -10.6443 | -12.4618 | -8.1249 | -7.6780 | -8.0374 | -9.2599 | -6.0961 | -7.9168 | -8.7824 | -10.5344 | -5.2991 |
| **ACS epi** | -10.2175 | -9.6813 | -12.5142 | -7.9208 | -7.6971 | -7.4180 | -8.5568 | -6.8374 | -6.5276 | -6.2452 | -9.1498 | -3.9054 |
| **ECS epi** | -9.7331 | -10.3758 | -12.9497 | -6.5165 | -8.4004 | -7.9822 | -9.9449 | -6.8559 | -8.0787 | -9.4783 | -10.9104 | -5.2469 |
| **BCS epi** | -11.5829 | -11.6097 | -13.3785 | -9.7874 | -8.6470 | -9.6507 | -11.8671 | -5.4270 | -9.7069 | -11.0469 | -13.4637 | -5.9501 |
| **GCSR epi** | .6019 | .6082 | .3685 | .8353 | .3128 | .3129 | .2458 | .3799 | .4546 | .3966 | .2977 | .6115 |
| **ACSR epi** | .7354 | .7366 | .6209 | .8498 | .5270 | .4869 | .4043 | .6498 | .7206 | .6583 | .4962 | .9449 |
| **ECSR epi** | .6325 | .6498 | .3102 | .9548 | .3918 | .3666 | .3079 | .4757 | .6210 | .5383 | .4157 | .8264 |
| **BCSR epi** | .9630 | .8696 | .7280 | 1.1980 | .4903 | .5628 | .3148 | .6659 | .7054 | .6551 | .4625 | .9483 |
| **GLS endo** | -24.4226 | -24.6814 | -28.8947 | -19.9504 | -18.7670 | -18.0128 | -22.7115 | -14.8224 | -20.2252 | -21.7369 | -27.6395 | -12.8108 |
| **GLSR endo** | 2.1421 | 2.0503 | 1.4475 | 2.8368 | 1.1618 | 1.0998 | .7612 | 1.5624 | 1.7178 | 1.6784 | 1.1530 | 2.2826 |
| **GLS epi** | -15.3865 | -15.6135 | -17.3431 | -13.4300 | -11.1170 | -10.9479 | -12.4308 | -9.8033 | -14.5839 | -14.6237 | -18.0582 | -11.1095 |
| **GLSR epi** | 1.2047 | 1.0325 | .5635 | 1.8460 | .7716 | .7558 | .6772 | .8661 | 1.2608 | 1.1882 | .9757 | 1.5459 |
| **LVEF** | 57.0000 | 57.0000 | 52.1116 | 61.8884 | 43.8750 | 45.0000 | 40.0980 | 47.6520 | 49.7500 | 50.0000 | 45.6604 | 53.8396 |

endo = endocardial, epi = epicardial, ACS = apical circumferential strain, ACSR = apical circumferential strain rate, BCS = basal circumferential strain, BCSR = basal circumferential strain rate, ECS = equatorial circumferential strain, ECSR = equatorial circumferential strain rate, GCS = global circumferential strain, GCSR = global circumferential strain rate, GLS = global longitudinal strain, GLSR = global longitudinal strain rate

Table S4: P-value results from non-parametric one-way ANOVA on ranks (Kruskal-Wallis) and post-hoc tests (Mann Whitney U). The green highlight denotes changes considered significant with p<0.002.

| **Variable** | **Kruskal-Wallis** | **Mann-Whitney** | **Mann-Whitney** | **Mann-Whitney** |
| --- | --- | --- | --- | --- |
|  |  | Baseline vs Acute | Acute vs Chronic | Baseline vs Chronic |
| GCS endo | 0.004 | 0.002 | 0.234 | 0.006 |
| ACS endo | 0.005 | 0.004 | 1.000 | 0.002 |
| ECS endo | 0.042 | 0.017 | 0.491 | 0.065 |
| BCS endo | 0.369 | 0.247 | 0.295 | 1.000 |
| GCSR endo | 0.018 | 0.002 | 0.234 | 0.171 |
| ACSR endo | 0.168 | 0.126 | 0.345 | 0.222 |
| ECSR endo | 0.054 | 0.009 | 0.228 | 0.354 |
| BCSR endo | 0.082 | 0.052 | 0.181 | 0.268 |
| GCS epi | 0.074 | 0.030 | 0.463 | 0.127 |
| ACS epi | 0.057 | 0.032 | 0.354 | 0.065 |
| ECS epi | 0.523 | 0.548 | 0.833 | 0.284 |
| BCS epi | 0.238 | 0.095 | 0.530 | 0.432 |
| GCSR epi | 0.022 | 0.010 | 0.121 | 0.127 |
| ACSR epi | 0.108 | 0.056 | 0.127 | 0.622 |
| ECSR epi | 0.085 | 0.151 | 0.030 | 0.943 |
| BCSR epi | 0.021 | 0.008 | 0.268 | 0.073 |
| LVEF | 0.002 | 0.002 | 0.028 | 0.019 |
| GLS endo | 0.026 | 0.008 | 0.181 | 0.171 |
| GLSR endo | 0.022 | 0.008 | 0.142 | 0.171 |
| GLS epi | 0.006 | 0.006 | 0.005 | 0.914 |
| GLSR epi | 0.013 | 0.012 | 0.014 | 0.762 |

**Table S5: Correlation between endocardial ECSR and most overexpressed proteins after MI**

| Protein | Infarcted vs Healthy myocardium | Correlation with ECSR | |
| --- | --- | --- | --- |
| Accession | Fold Change | R-squared | p-value |
| A5GFY8 | 2.027 | 0.96 | 0.01 |
| I3LB66 | 2.39 | 0.96 | 0.01 |
| F1RYJ8 | 2.42 | 0.95 | 0.02 |
| F1RVS9 | 2.65 | 0.94 | 0.02 |
| F1SCR9 | 2.09 | 0.94 | 0.02 |
| Q6EEI0 | 2.66 | 0.94 | 0.02 |
| F1RF27 | 3.63 | 0.93 | 0.03 |
| F1RF28 | 2.36 | 0.93 | 0.03 |
| F1S5Q1 | 2.6 | 0.93 | 0.03 |
| I3L7W9 | 2.01 | 0.93 | 0.03 |
| F1RPQ0 | 2.15 | 0.92 | 0.03 |
| F1SLT8 | 2.19 | 0.92 | 0.03 |
| F1SSF7 | 2.52 | 0.92 | 0.03 |
| F1S1D2 | 2.81 | 0.91 | 0.04 |
| F1S6B5 | 4.02 | 0.91 | 0.04 |
| F1SJL4 | 2.26 | 0.91 | 0.04 |
| Q29116 | 2.9 | 0.91 | 0.04 |
| F1RQI0 | 5.29 | 0.9 | 0.04 |
| F1RQI2 | 12.1 | 0.9 | 0.04 |
| F1RIP3 | 2.29 | 0.9 | 0.05 |
| I3LPW3 | 2.63 | 0.89 | 0.05 |
| B3F0B7 | 2.13 | 0.86 | 0.06 |

ECSR= *equatorial circumferential strain rates*, MI= *Myocardial Infarction*

**Table S6: Linear Models and relative p-values for correlation between Strain Rates and most overexpressed proteins in infarcted area**

|  | Damage vs Healthy | Global Strain Rate | | Longitudinal Global Strain Rate | | Basal Strain Rate | | Equatorial Strain Rate | | Apical Strain Rate | |
| --- | --- | --- | --- | --- | --- | --- | --- | --- | --- | --- | --- |
| Protein |  |  | |  | |  | |  | |  | |
| Accession | Fold Change | R-squared | p- value | R-squared | p- value | R-squared | p-value | R-squared | p-value | R- squared | p-value |
| A5GFY8 | 2.027 | 0.34 | 0.4 | 0.03 | 0.82 | 0.22 | 0.52 | 0.96 | 0.01 | 0.0007 | 0.97 |
| A5YV76 | 2.076 | 0.23 | 0.51 | 0.94 | 0.03 | 0.39 | 0.37 | 0.01 | 0.88 | 0.09 | 0.68 |
| B1VD75 | 4.49 | 0.26 | 0.48 | 0.95 | 0.02 | 0.36 | 0.39 | 0.02 | 0.84 | 0.1 | 0.67 |
| B3F0B7 | 2.13 | 0.3 | 0.44 | 0.13 | 0.63 | 0.03 | 0.82 | 0.86 | 0.06 | 0.01 | 0.86 |
| F1RF13 | 2.62 | NA | NA | NA | NA | NA | NA | NA | NA | NA | NA |
| F1RF27 | 3.63 | 0.31 | 0.43 | 0.06 | 0.75 | 0.11 | 0.65 | 0.93 | 0.03 | 0.009 | 0.9 |
| F1RF28 | 2.36 | 0.38 | 0.38 | 0.13 | 0.63 | 0.06 | 0.73 | 0.93 | 0.03 | 0.002 | 0.95 |
| F1RIP3 | 2.29 | 0.36 | 0.39 | 0.16 | 0.59 | 0.03 | 0.8 | 0.9 | 0.05 | 0.004 | 0.93 |
| F1RJ12 | 2.71 | 0.04 | 0.78 | 0.21 | 0.53 | 0.07 | 0.72 | 0.25 | 0.49 | 0.5 | 0.29 |
| F1RPQ0 | 2.15 | 0.31 | 0.43 | 0.07 | 0.72 | 0.09 | 0.69 | 0.92 | 0.03 | 0.01 | 0.89 |
| F1RQG9 | 2.18 | NA | NA | NA | NA | NA | NA | NA | NA | NA | NA |
| F1RQI0 | 5.29 | 0.34 | 0.41 | 0.13 | 0.64 | 0.04 | 0.78 | 0.9 | 0.04 | 0.008 | 0.9 |
| F1RQI2 | 12.1 | 0.34 | 0.41 | 0.13 | 0.63 | 0.04 | 0.78 | 0.9 | 0.04 | 0.008 | 0.9 |
| F1RQM4 | 2.08 | 0.18 | 0.57 | 0.89 | 0.05 | 0.44 | 0.33 | 0.0004 | 0.97 | 0.1 | 0.67 |
| F1RT92 | 2.83 | 0.25 | 0.66 | 0.19 | 0.71 | 0.21 | 0.69 | 0.88 | 0.21 | 0.05 | 0.84 |
| F1RVS9 | 2.65 | 0.4 | 0.36 | 0.14 | 0.63 | 0.07 | 0.72 | 0.94 | 0.02 | 0.0005 | 0.97 |
| F1RYJ8 | 2.42 | 0.42 | 0.34 | 0.15 | 0.61 | 0.08 | 0.71 | 0.95 | 0.02 | 0.000001 | 0.99 |
| F1S1B2 | 2.44 | 0.14 | 0.62 | 0.81 | 0.09 | 0.44 | 0.33 | 0.01 | 0.87 | 0.15 | 0.6 |
| F1S1D2 | 2.81 | 0.33 | 0.42 | 0.1 | 0.67 | 0.06 | 0.74 | 0.91 | 0.04 | 0.01 | 0.89 |
| F1S5Q1 | 2.6 | 0.32 | 0.42 | 0.05 | 0.92 | 0.33 | 0.41 | 0.93 | 0.03 | 0.0000003 | 0.99 |
| F1S6B5 | 4.02 | 0.35 | 0.43 | 0.09 | 0.7 | 0.07 | 0.72 | 0.91 | 0.04 | 0.01 | 0.89 |
| F1SCR9 | 2.09 | 0.49 | 0.29 | 0.21 | 0.54 | 0.06 | 0.74 | 0.94 | 0.02 | 0.003 | 0.94 |
| F1SEN5 | 2.38 | 0.008 | 0.9 | 0.09 | 0.69 | 0.2 | 0.54 | 0.54 | 0.26 | 0.21 | 0.53 |
| F1SFT6 | 3.11 | 0.13 | 0.62 | 0.82 | 0.09 | 0.46 | 0.31 | 0.01 | 0.89 | 0.13 | 0.62 |
| F1SIJ9 | 3.76 | 0.002 | 0.96 | 0.68 | 0.38 | 0.85 | 0.24 | 0.02 | 0.89 | 0.11 | 0.77 |
| F1SJL4 | 2.26 | 0.38 | 0.37 | 0.16 | 0.59 | 0.04 | 0.78 | 0.91 | 0.04 | 0.002 | 0.94 |
| F1SLT8 | 2.19 | 0.35 | 0.4 | 0.11 | 0.67 | 0.07 | 0.72 | 0.92 | 0.03 | 0.005 | 0.92 |
| F1SN73 | 2.15 | NA | NA | NA | NA | NA | NA | NA | NA | NA | NA |
| F1SSF7 | 2.52 | 0.32 | 0.42 | 0.09 | 0.7 | 0.08 | 0.71 | 0.92 | 0.03 | 0.009 | 0.9 |
| F2Z5P9 | 2.02 | 0.07 | 0.72 | 0.09 | 0.7 | 0.009 | 0.96 | 0.18 | 0.57 | 0.68 | 0.17 |
| I3L7W9 | 2.01 | 0.29 | 0.45 | 0.04 | 0.81 | 0.15 | 0.6 | 0.93 | 0.03 | 0.01 | 0.89 |
| I3LB66 | 2.39 | 0.43 | 0.34 | 0.12 | 0.64 | 0.1 | 0.66 | 0.96 | 0.01 | 0.0002 | 0.98 |
| I3LE10 | 2.93 | 0.94 | 0.02 | 0.57 | 0.24 | 0.05 | 0.75 | 0.64 | 0.19 | 0.34 | 0.4 |
| I3LL43 | 2.26 | 0.98 | 0.006 | 0.42 | 0.34 | 0.16 | 0.58 | 0.65 | 0.19 | 0.45 | 0.32 |
| I3LPW3 | 2.63 | 0.24 | 0.5 | 0.03 | 0.81 | 0.11 | 0.66 | 0.89 | 0.05 | 0.03 | 0.82 |
| Q29116 | 2.9 | 0.38 | 0.37 | 0.16 | 0.59 | 0.04 | 0.78 | 0.91 | 0.04 | 0.002 | 0.94 |
| Q2TCH2 | 2.4 | 0.25 | 0.49 | 0.94 | 0.03 | 0.37 | 0.38 | 0.01 | 0.86 | 0.1 | 0.67 |
| Q5S1S4 | 4.1 | 0.26 | 0.48 | 0.95 | 0.02 | 0.36 | 0.39 | 0.02 | 0.84 | 0.1 | 0.67 |
| Q6EEI0 | 2.66 | 0.32 | 0.43 | 0.05 | 0.76 | 0.12 | 0.64 | 0.94 | 0.02 | 0.007 | 0.91 |

**Table S7.** Correlation analysis of GCS, GLS, ACS with LVEF and ESVi

| Strain | LVEF | | ESVi | |
| --- | --- | --- | --- | --- |
|  | r | p | r | p |
| GCS | -0.73 | 0.0002* | -0.35 | 0.12 |
| ACS | -0.60 | 0.004* | -0.14 | 0.55 |
| GLS | -0.73 | 0.001* | -0.32 | 0.12 |

*Correlation analysis of GCS, GLS, ACS with LVEF and ESVi using the data from corresponding time points. Correlations between endocardial mechanical strain data and volumetric clinical indices. * denotes significance to p ≤0.05.*

**Supplementary Figures**

***Figure S1***


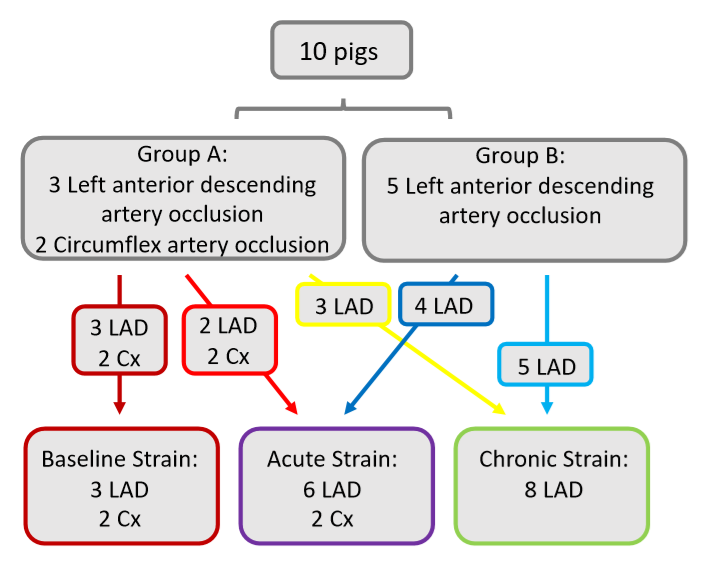


*Flow chart showing data contribution from the 10 pigs for the calculation of basal, acute and chronic strain.*

***Figure S2***

*
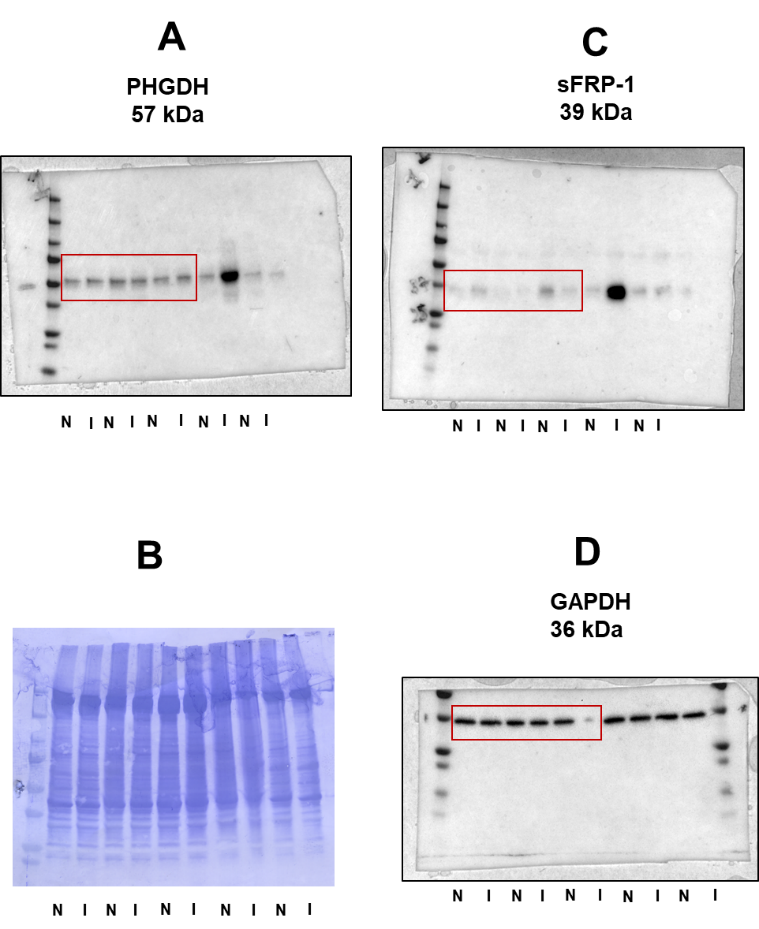
*

*Full blots for the proteins shown in Fig. 6. The bands of each protein shown in Fig. 6 are marked by the red rectangular frame. Optical density for the Western blot of PHDH (panel A) was normalised for total protein on the PVDF membrane stained with Coomassie Blue (panel B). Optical density for the Western blot of sFRP-1 (panel C) was normalised for the Western blot of GAPDH (panel D). N – non-ischaemic myocardium; I – infarcted myocardium.*

**Supplementary Videos**

**Video 1:**

Cinematic image in three-chamber plane at baseline time point demonstrating non-dilated left ventricular cavity with normal contraction of all walls seen.

**Video 2:**

Cinematic image in three-chamber plane at acute time point demonstrating dilatation of the left ventricular cavity with severe hypokinesia of the mid to apical anteroseptal segments.

**Video 3:**

Cinematic image in three-chamber plane at chronic time point demonstrating further dilatation of the left ventricular cavity with thinning and akinesia of the mid to apical anteroseptal segments.
